# Efficacy and safety evaluation of Azvudine in the prospective treatment of COVID-19 based on four phase III clinical trials

**DOI:** 10.3389/fphar.2023.1228548

**Published:** 2023-08-24

**Authors:** Ke-Wei Zhu

**Affiliations:** Office of Pharmacovigilance, Guangzhou Baiyunshan Pharmaceutical Holding Co., Ltd., Baiyunshan Pharmaceutical General Factory, Guangzhou, Guangdong, China

**Keywords:** Azvudine (FNC), viral load, severe acute respiratory syndrome coronavirus 2, RNA-dependent RNA polymerase, nucleic acid-negative conversion time

## Abstract

Azvudine (FNC) is a synthetic nucleoside analog used to treat adult patients living with human immunodeficiency virus-1 (HIV-1) infection with high viral load. After phosphorylation, Azvudine inhibits RNA-dependent RNA polymerase, leading to the discontinuation of RNA chain synthesis in viruses. In addition, Azvudine is the first dual-target nucleoside oral drug worldwide to simultaneously target reverse transcriptase and viral infectivity factors in the treatment of HIV infection. On 9 August 2022, Azvudine was incorporated into the Guidelines for the Diagnosis and Treatment of Coronavirus Disease 2019 (version ninth) issued by the National Health Commission and the National Administration of Traditional Chinese Medicine. The recommended oral dose of Azvudine for the treatment of moderate coronavirus disease 2019 (COVID-19) is 5 mg once daily, and the duration of Azvudine treatment should not exceed 14 days. Four phase III clinical trials were performed during 2020–2022 to evaluate the efficacy and safety of Azvudine in the treatment of COVID-19. The results revealed that Azvudine could reduce nucleic acid-negative conversion time, viral load, and time to improvement in clinical conditions in patients with moderate COVID-19. In addition, Azvudine exhibited good safety and tolerance. Thereafter, Azvudine was incorporated into the Chinese guidelines and expert consensus for the treatment of COVID-19 and was highly approbated. Furthermore, Azvudine was also included in the Chinese guidelines for HIV infection.

## 1 Introduction

Azvudine (FNC) is a synthetic nucleoside analog used to treat adult patients with plasma human immunodeficiency virus-1 (HIV-1) RNA of more than 100,000 copies/mL through the combination of another nucleoside reverse transcriptase inhibitor (NRTI) or non-nucleoside reverse transcriptase inhibitor (NNRTI). Azvudine inhibits RNA-dependent RNA polymerase (RdRp) after phosphorylation, leading to the discontinuation of RNA chain synthesis in viruses, thereby playing an antiviral role (Henan Genuine Biotech, 2022). On 21 July 2021, Azvudine (Shuangxinaike^®^, Henan Genuine Biotech Co., Ltd., Pingdingshan, Henan Province, China) was approved by the National Medical Products Administration (NMPA, Beijing, China) for the treatment of adult HIV-1-infected patients (National Medical Products Administration, 2021). In addition, Azvudine is the first dual-target nucleoside oral agent worldwide to simultaneously target both reverse transcriptase and viral infectivity factors for the treatment of HIV infection. As a Chinese homegrown innovative drug for the treatment of acquired immunodeficiency syndrome (AIDS), Azvudine was developed with funding from the National Science and Technology Major Projects for Major New Drug Innovation (Genuine Biotech, 2021). Thereafter, Azvudine was incorporated into the Chinese guidelines for the diagnosis and treatment of AIDS (Acquired Immunodeficiency Syndrome and Hepatitis C Professional Group, Society of Infectious Diseases, Chinese Medical Association and Chinese Center for Disease Control and Prevention, 2021).

The ChemSrc database (https://www.chemsrc.com/en) was used to inquire about the chemical structure of Azvudine. The molecular formula of Azvudine is C_9_H_11_FN_6_O_4_ with a molecular weight of 286.22, and the chemical name is 4-amino-1-[(2R,3S,4R,5R)-5-azido-3-fluoro-4-hydroxy-5-(hydroxymethyl)tetrahydro-2-furanyl]-2(1H)-pyrimidinone (Supplementary Figure S1).

RdRp is highly conserved in the severe acute respiratory syndrome coronavirus 2 (SARS-CoV-2) and is an excellent target for the development of anti-SARS-CoV-2 agents (Rabie, 2021a; Rabie, 2021b; Rabie, 2021c; Vicenti et al., 2021; Rabie et al., 2023; Zhu, 2023a). Nucleoside analogs were considered candidate drugs for the treatment of coronavirus disease 2019 (COVID-19), and several nucleoside analogs were identified as promising anti-SARS-CoV-2 drugs based on satisfactory results of preclinical trials (Rabie and Abdalla, 2022; Eltayb et al., 2023; Rabie and Abdalla, 2023). Nucleoside analogs targeting RdRp are ideal anti-SARS-CoV-2 candidate drugs in consideration of the broad conservation of RdRp in all SARS-CoV-2 variants (Rabie, 2022a; Rabie, 2022b; Rabie and Abdalla, 2023; Rabie and Eltayb, 2023). Azvudine, as a nucleoside analog targeting highly conserved RdRp, had potential anti-SARS-CoV-2 effects and was selected as a possible effective candidate drug for the treatment of COVID-19 (Yu and Chang, 2020). A preclinical trial revealed that Azvudine was transformed into active Azvudine triphosphate concentrated in the thymus after phosphorylation, inhibited SARS-CoV-2 replication, and improved immunity. The subsequent pilot clinical study of Azvudine exhibited its excellent efficacy and satisfactory safety in the treatment of COVID-19 (Zhang et al., 2021). In addition, Ren et al. (2020) performed a preliminary clinical trial of Azvudine with a randomized, open-label, controlled design. A total of 20 patients with mild-to-moderate COVID-19 were randomly assigned to receive Azvudine or standard antiviral treatment with a ratio of 1:1. The results revealed that Azvudine shortens the time of nucleic acid-negative conversion (NANC) compared to standard antiviral treatment. Moreover, Azvudine displayed satisfactory safety without adverse events occurring during the treatment.

However, the two pilot clinical studies of Azvudine are short of a large sample size. Thereafter, four phase III clinical trials were performed during 2020–2022 to evaluate the efficacy and safety of Azvudine in the treatment of COVID-19. Here, the therapeutic effects of Azvudine on COVID-19 were comprehensively analyzed using the clinical data from four phase III clinical trials.

## 2 Four phase III clinical trials

### 2.1 A phase III clinical trial in China

The four phase III clinical trials were carried out in China, Russia, and Brazil. In China, the phase III clinical trial was registered in the Chinese Clinical Trial Registry (ChiCTR2000032769; registration date: 9 May 2020; website: https://www.chictr.org.cn/showproj.aspx?proj=53368), and the clinical trial was simultaneously registered in the International Clinical Trials Registry Platform (NCT04425772; registration date: 9 May 2020; website: https://trialsearch.who.int/Trial2.aspx?TrialID=NCT04425772). The randomized, double-blinded, parallel-controlled clinical trial was carried out in Beijing Ditan Hospital affiliated to Capital Medical University (Beijing, China) from June 2020 to March 2022. The sponsor was Henan Genuine Biotech Co., Ltd. A total of 348 patients with COVID-19 were enrolled in the study, and the patients were randomly assigned to the Azvudine group or control group with a 1:1 ratio. The patients in the Azvudine group received oral Azvudine tablets (5 mg once daily) plus standard treatment, and the patients in the control group received Azvudine dummy tablets (placebo) and standard treatment. The duration of treatment in both groups was less than 14 days. The primary efficacy outcome was the change (reduction) from baseline in viral load on days 7 and 14. The secondary primary efficacy outcomes included the time of NANC, nucleic acid conversion rate, the proportion of patients changing from mild-or-moderate COVID-19 to severe COVID-19 in the severity of the disease, the proportion of patients changing from severe COVID-19 to critical COVID-19 in severity, time and proportion of improvement in pulmonary imaging, time and rate of improvement in respiratory symptoms and other symptoms, frequency of requirement for supplemental oxygen or non-invasive ventilation, changes in blood oxygen detection index, and time and proportion of temperature return to normal levels. As a result, among the patients with viral load ≥ 3 log_10_ copies/mL, the reduction from baseline in viral load on days 3, 5, and 7 in the Azvudine group was higher than that in the placebo group. However, only the difference in the reduction from baseline in viral load on day 5 between the Azvudine group and placebo group was statistically significant. This result was similar to that in the patients with viral load ≥ 4 log_10_ copies/mL. Unfortunately, there were no significant differences in secondary primary efficacy outcomes between the Azvudine and placebo groups. In addition, 341 subjects were involved in the safety analysis. A total of 62 subjects experienced 119 adverse events (AEs) in the Azvudine group, while 76 subjects experienced 175 AEs in the placebo group. Most of the AEs were evaluated as grade 1 or grade 2 according to the Common Terminology Criteria for Adverse Events (CTCAE) version 4.03 of the National Cancer Institute. One patient experienced an AE that was evaluated as grade 3 in the Azvudine group, while three patients experienced three AEs that were evaluated as grade 3 in the placebo group. No AEs were evaluated as grade 4, or no serious AEs occurred. There was no significant difference in the incidence or severity of AEs between the Azvudine and placebo groups (ClinicalTrials, 2022; Zhang, 2022).

### 2.2 A phase III clinical trial in Russia

In Russia, the phase III clinical trial was approved by the Russian Ministry of Health (http://www.minzdravsoc.ru) in January 2021 and was initiated in June 2021. The results of the two phase III clinical trials performed in China and Russia were disclosed in detail by Fujie Zhang at the 17th National Infectious Diseases Conference, which was hosted by the Chinese Medical Association in Hefei (Anhui, China) during 11-13 August 2022 (Chinese Society of Infectious Diseases and Chinese Medical Association, 2022). Fujie Zhang, a chief physician at Beijing Ditan Hospital affiliated with Capital Medical University (Beijing, China), was also the principal investigator (PI) in the Chinese phase III clinical trial. In addition, the results of the phase III clinical trial performed in Russia were recorded in the package insert of Azvudine tablets, manufactured by Henan Genuine Biotech Co., Ltd., and presented in Chinese language (Henan Genuine Biotech, 2022). A total of 314 patients with moderate COVID-19 were enrolled as the full analysis set (FAS) in the study, and the patients were randomly assigned to the Azvudine group or control group with a 1:1 ratio. The primary efficacy outcome was the median time to improvement in clinical conditions and the proportion of improvement in clinical conditions on day 7. A total of 279 patients were eligible according to inclusion and exclusion criteria and were included in the per-protocol set (PPS), which consisted of 141 subjects in the Azvudine group and 138 subjects in the control group. The mean baseline age was 48 years, with 39% and 61% of subjects falling under age groups 18–45 years (inclusive of 18 years) and 45–65 years (inclusive of 45 years), respectively. Male and female subjects accounted for 43% and 57%, respectively. There was no significant difference in the demographic or clinical characteristics between the Azvudine and control groups. As shown in Table 1, Azvudine significantly elevated the proportion of improvement in clinical conditions on day 7 and reduced the median time to improvement in clinical conditions compared to placebo in both FAS and PPS (Henan Genuine Biotech, 2022). With regard to safety, 34 patients underwent 47 AEs in the Azvudine group, and 35 patients underwent 50 AEs in the placebo group. Most of the AEs were determined as grade 1 or grade 2 according to CTCAE version 4.03. Only one serious AE occurred in the placebo group, and no serious AE occurred in the Azvudine group. There was no statistically significant difference in the frequency or severity of AEs between the Azvudine and placebo groups (ClinicalTrials, 2022; Zhang, 2022).

**TABLE 1:** Median time to improvement in clinical conditions and the proportion of improvement in clinical conditions on day 7 after the administration of Azvudine and placebo.

Parameters	Full analysis set		Per protocol set	
	Azvudine	Placebo	Azvudine	Placebo
N (missing)	157 (0)	157 (0)	141 (0)	138 (0)
Endpoint (%)	138 (87.90%)	136 (86.62%)	137 (97.16%)	136 (98.55%)
Censoring (%)	19 (12.10%)	21 (13.38%)	4 (2.84%)	2 (1.45%)
Improvement	57 (36.31%)	15 (9.55%)	57 (40.43%)	15 (10.87%)
No improvement	100 (63.69%)	142 (90.45%)	84 (59.57%)	123 (89.13%)
*p*-value	<0.001		<0.001	
Median time	10.00	13.00	10.00	13.00
*p*-value	<0.001		<0.001	

^a^ The clinical data were obtained from the package insert of Azvudine tablets manufactured by Henan Sincere Biotechnology Co., Ltd.

^b^ The missing values in the primary efficacy outcome were imputed using the worst value method.

^c^ Clinical conditions were determined according to the WHO Ordinary Clinical Progression Scale (Jun/2020), score of 4 to 10. The data were acquired according to the WHO score between day 1 and day 31.

^d^ The time to improvement in clinical conditions was calculated in terms of the first time improvement in clinical conditions emerged, which was determined by a decrease of ≥ 2 in the WHO score. Patients without any improvement in clinical condition by the end of the study were denoted as censoring patients. The last time that the WHO score was determined was used to calculate the censoring time.

### 2.3 Two phase III clinical trials in Brazil

In Brazil, two phase III clinical trials of Azvudine were registered on the International Clinical Trials Registry Platform (NCT05033145 and NCT04668235). One trial was performed on patients with mild COVID-19, and the other one was performed on patients with moderate COVID-19. In the phase III clinical trial performed on patients with mild COVID-19, the primary outcome was the proportion of patients hospitalized within 4 weeks after randomization. The secondary outcomes involved the negative conversion time of the SARS-CoV-2 viral load, the proportion of patients cured during treatment, the severity and duration of COVID-19 symptoms, and the duration of Azvudine treatment until the second negative conversion. Patients with mild COVID-19 were randomly assigned to receive Azvudine 10 mg once a night or a placebo. Additionally, all the patients received standard treatment. A total of 281 patients with mild COVID-19 were included in the analysis (da Silva et al., 2023). For the phase III clinical trial performed in patients with moderate COVID-19, the primary outcome was the proportion of patients with improved clinical status. The secondary outcomes included the proportion of patients with a clinical outcome of cure after treatment, time to improvement in COVID-19 symptoms, hospital length of stay (LOS), and negative conversion time of the SARS-CoV-2 viral load. Patients with moderate COVID-19 were randomly assigned with a ratio of 1:1 to receive Azvudine 10 mg once a night plus standard treatment or placebo plus standard treatment. A total of 180 eligible patients with moderate COVID-19 were enrolled, of whom 172 completed the study (Cabral et al., 2022). The results of both phase III clinical trials revealed that Azvudine significantly reduced the NANC time and viral load of the patients compared to the placebo group (da Silva et al., 2023; Cabral et al., 2022).

#### 2.3.1 Permission to reuse and copyright

The figures in this paper and Supplementary Material were re-plotted by the author based on the published data, and there is no third-party illustrative material in the report. The clinical data were obtained from package inserts of drugs, news reports, published conference reports, preprints, and published studies, which are publicly available sources.

### 2.4 Significant contributions to the fight against the COVID-19 pandemic by the end of 2022

The State Council of the People’s Republic of China (http://english.www.gov.cn), also known as the Chinese central government, announced 10 new measures for the COVID-19 response on 7 December 2022. Henceforth, the dynamic zero-COVID-19 policy was abrogated (Xinhua, 2022a; Zhu, 2023). Without the restrictions of quarantine measures, SARS-CoV-2 Omicron variants rapidly spread across the Chinese mainland and caused large-scale infections. To date, only two anti-SARS-CoV-2 agents were approved by the NMPA for the treatment of COVID-19, and another one was nirmatrelvir–ritonavir (Paxlovid^®^, Pfizer, New York, State of New York, USA). NMPA granted conditional approval for the imports of Paxlovid on 11 February 2022 (Xinhua, 2022b). However, the price of Paxlovid was too high relative to Azvudine in the Chinese market. The prices of Paxlovid and Azvudine in medical insurance were 2,300 Yuan per box and 270 Yuan per bottle, respectively. Therefore, the preferred antiviral drug was Azvudine instead of Paxlovid for Chinese people during the COVID-19 pandemic. In this situation, numerous domestic public hospitals introduced Azvudine to cope with the current SARS-CoV-2 Omicron wave. A total of 113 community hospitals admitted Azvudine as a prescription drug in the treatment of COVID-19 on 3 January 2023. Genuine Biotech, together with Shanghai Fosun Pharmaceutical (Group) Co., Ltd., donated 100 million Yuan’s worth of Azvudine to the rural areas of Midwestern China, covering 180 counties, in several phases through the Shanghai Fosun Foundation on 9 January 2023 (Genuine Biotech, 2023a). In fact, patients with COVID-19 in rural and remote areas should be given more attention due to poor medical conditions and health outcomes (Zhu, 2023). Within 48 h of the announcement, the first batch of 6,000 bottles of Azvudine tablets was conveyed to 10 county-/village-level clinics in four provinces through human-powered transportation. As of 28 February 2023, the two pharmaceutical companies had donated nearly 300,000 bottles of Azvudine tablets to Chinese rural areas, covering 250 cities/counties in nine provinces and the Shanghai municipality (Genuine Biotech, 2023b). Thereafter, in the award ceremony for “Henan Socially Responsible Enterprise of 2022 and Entrepreneur with Outstanding Social Contribution,” Genuine Biotech became the winner after several rounds of selection and was honored with the “2022 Annual Award of Henan Socially Responsible Enterprise” (Genuine Biotech, 2023c).

## 3 Discussion

In summary, Azvudine could decrease the time of NANC, viral load, and the median time for improvement in clinical conditions and increase the proportion of improvement in clinical conditions on day 7 in patients with moderate COVID-19. Additionally, Azvudine could reduce body temperature normalization time, fever duration, and hospital LOS. In safety analyses, Azvudine exhibited good safety and tolerance. In summary, Azvudine was effective and well tolerated in the treatment of moderate COVID-19.

However, there were some limitations in the four phase III trials. In the trial performed in China, there were no significant differences between the Azvudine and control groups for most of the clinical outcomes; only the difference in the reduction in viral load from baseline on day 5 between the Azvudine and placebo groups was statistically significant. Azvudine did not display effectiveness in the alleviation of respiratory symptoms and prevention of progression to severe or critical COVID-19. For the phase III clinical trial performed in Brazilian patients with mild COVID-19, there also were no significant differences between Azvudine and placebo for most of the clinical outcomes, especially the primary outcome, and the researchers attributed the failure to the self-limiting nature of mild COVID-19 (da Silva et al., 2023). In the phase III clinical trial performed on Brazilian patients with moderate COVID-19, Azvudine significantly improved the clinical status at the time of discharge and reduced body temperature normalization time, fever duration, and hospital LOS compared to placebo (Cabral et al., 2022). The two phase III clinical trials in Brazil suggested Azvudine exhibited good efficacy in patients with moderate COVID-19 instead of in patients with mild COVID-19. In the four phase III clinical trials, whether Azvudine could reduce COVID-19 mortality or prevent progression to severe/critical COVID-19 was unclear and remained a major concern. Moreover, the sample sizes in the four phase III clinical trials were relatively small as compared to other high-quality phase III clinical trials of anti-SARS-CoV-2 drugs (Hammond et al., 2022; Jayk Bernal et al., 2022; Cao et al., 2023). Nevertheless, the four phase III clinical trials have demonstrated that Azvudine effectively reduced the time of NANC and viral load in patients with COVID-19.

**FIGURE 1:**
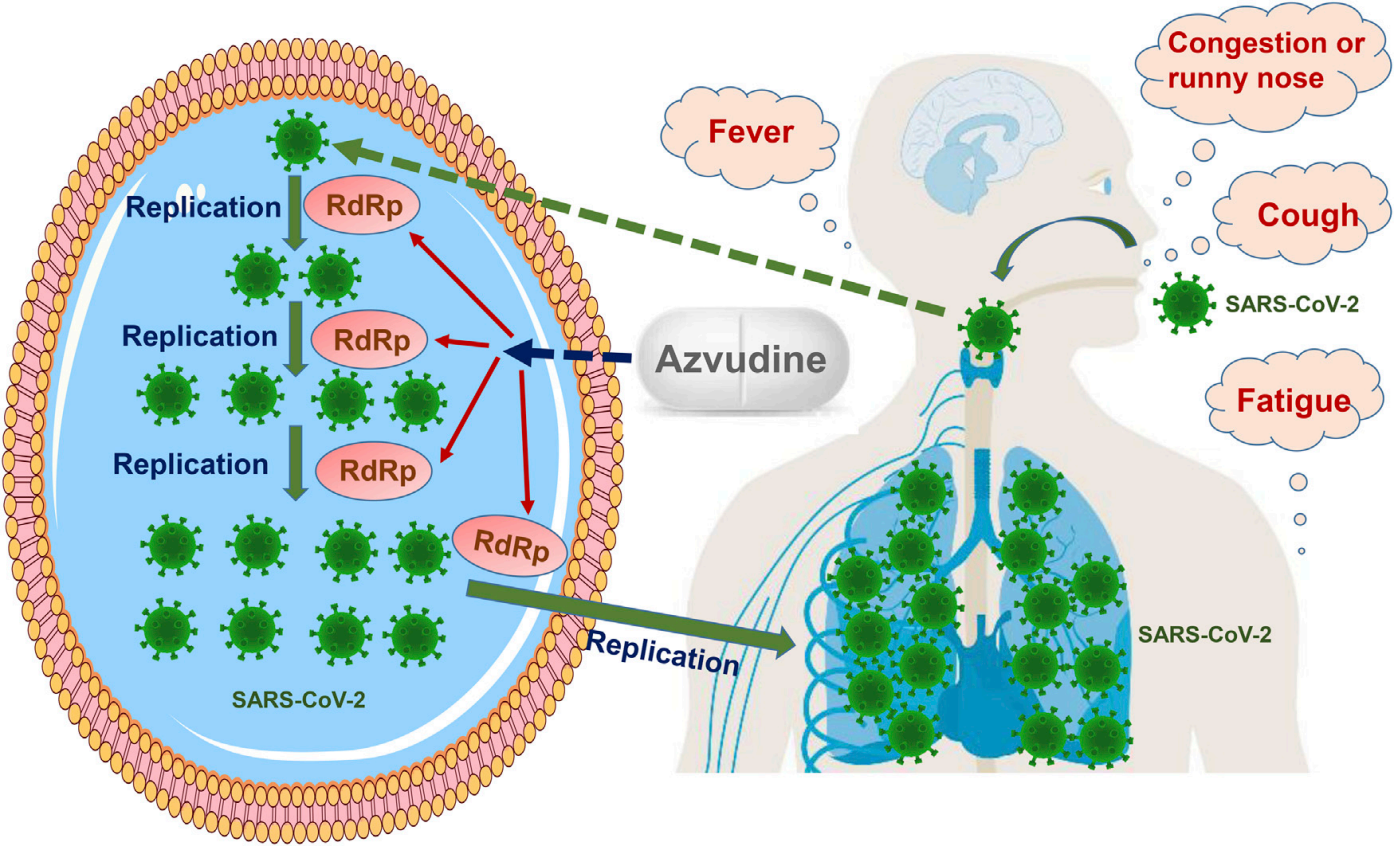
Therapeutic effect of Azvudine on COVID-19. Azvudine inhibits RNA-dependent RNA polymerase (RdRp), which is a vital enzyme mediating SARS-CoV-2 replication and transcription. Azvudine plays an anti-SARS-CoV-2 role by blocking virus replication.

After entering a host cell, the genomic RNA of SARS-CoV-2 acts as a template and is translated to yield two polyproteins by utilizing the host cell protein synthesis system, followed by proteolytic cleavage with two cysteine proteases to form a replication–transcription complex mediating SARS-CoV-2 RNA synthesis, capping, and proofreading. New SARS-CoV-2 genomic RNA is translated to produce additional non-structural proteins, which are used for further RNA synthesis or new virion assembly. Then, SARS-CoV-2 genomic RNA is coated with nucleocapsid proteins to generate nucleocapsid structures. Lipid bilayers consisting of viral spikes, membranes, and envelope proteins are synthesized in the endoplasmic reticulum–Golgi intermediate compartment. A mature progeny SARS-CoV-2 virion emerges after assembly and is released through exocytosis. Eventually, numerous SARS-CoV-2 virions are produced after several cycles of replication, leading to another round of infection (Malone et al., 2022; Yang and Rao, 2021; V'Kovski et al., 2021). The degree of infection is related to the innate antiviral response, and disease severity can promote the enhancement of the antibody response, which is associated with clinical outcomes. The entry of SARS-CoV-2 through the respiratory tract tends to cause the involvement of the upper and lower respiratory tracts, leading to a series of respiratory or systemic symptoms, such as cough, fever, and fatigue (Attaway et al., 2021; Gattinoni et al., 2021) (Figure 1).

RdRp is a vital enzyme that controls SARS-CoV-2 replication and transcription by catalyzing the phosphodiester binding process for RNA synthesis and is identified as a promising molecular target in COVID-19 (Tian et al., 2021; Ng et al., 2022). Azvudine, as an RdRp inhibitor, plays an anti-SARS-CoV-2 role by blocking virus replication (Figure 1). It should be noted that Azvudine has no role in the clearance of SARS-CoV-2, and it might be the fundamental reason that Azvudine did not exhibit effectiveness for several clinical outcomes in the four phase III clinical trials. On the other hand, in order to control viral load and prevent progression to severe COVID-19, Azvudine should be administered as soon as possible after the diagnosis of COVID-19 in accordance with the package insert of Azvudine tablets (Henan Genuine Biotech, 2022).

COVID-19 was added to the Azvudine list of indications in the package insert to resist the COVID-19 Omicron wave, and this application was conditionally approved by the NMPA on 25 July 2022 (National Medical Products Administration, 2022). On 9 August 2022, the National Health Commission and the National Administration of Traditional Chinese Medicine announced the inclusion of Azvudine in the Guidelines for the Diagnosis and Treatment of Coronavirus Disease 2019 (version ninth). The recommended oral dose of Azvudine for the treatment of moderate COVID-19 is 5 mg once daily, and the duration of Azvudine treatment should not exceed 14 days (National Health Commission of the People’s Republic of China and National Administration of Traditional Chinese Medicine, 2022; None, 2022). Azvudine was incorporated into the Guidelines for the Diagnosis and Treatment of Coronavirus Disease 2019 (version 10th) issued by the National Health Commission and National Administration of Traditional Chinese Medicine on 6 January 2023 (General Office of National Health Commission of the People’s Republic of China and General comprehensive affairs department of national administration of traditional Chinese medicine of the People’s Republic of China, 2023). Thereafter, Azvudine was included in the Expert Consensus on Antiviral Therapy for COVID-19, which was compiled by 34 Chinese experts, and was highly approbated (Zhang and Li, 2023). On 18 January 2023, Azvudine was officially included in the National Reimbursement Drug List and was priced at 11.58 Yuan/tablet/3 mg in medical insurance (Genuine Biotech, 2023d).

## 4 Conclusion and therapeutic applications

In patients with moderate COVID-19, Azvudine could reduce NANC time, viral load, and time to improvement in clinical conditions and exhibited good safety and tolerance. Azvudine is a promising anti-SARS-CoV-2 drug. However, randomized controlled trials with large sample sizes or large-scale real-world clinical studies are needed to evaluate the efficacy of Azvudine in preventing disease progression and reducing mortality in patients with COVID-19.

## Data Availability

The original contributions presented in the study are included in the article/Supplementary material; further inquiries can be directed to the corresponding author.
