# Supplementary material for: Efficacy and safety evaluation of Azvudine in the prospective treatment of COVID-19 based on four phase III clinical trials
Source: Front Pharmacol. 2023 Aug 24;14:1228548. doi: 10.3389/fphar.2023.1228548 (PMC10484631; doi:10.3389/fphar.2023.1228548)

Supplementary Material

Efficacy and safety evaluation of Azvudine in the treatment of COVID-19 based on four phase III clinical trials.

Ke-Wei Zhu*

*** Correspondence:** Ke-Wei Zhu: zhukew2018@outlook.com

1 Supplementary Figures and Tables

1.1 Supplementary Figures

Supplementary Figure S1. Chemical structure of Azvudine.

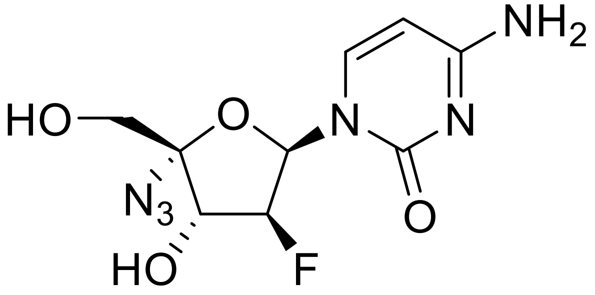

Supplement: Supplementary file 1 [file DataSheet1.pdf]
